# MegaSNPHunter: a learning approach to detect disease predisposition SNPs and high level interactions in genome wide association study

**DOI:** 10.1186/1471-2105-10-13

**Published:** 2009-01-09

**Authors:** Xiang Wan, Can Yang, Qiang Yang, Hong Xue, Nelson LS Tang, Weichuan Yu

**Affiliations:** 1 Department of Electronic and Computer Engineering, Hong Kong University of Science and Technology, Hong Kong, PR China; 2 Department of Computer Science, Hong Kong University of Science and Technology, Hong Kong, PR China; 3 Department of Biochemistry, Hong Kong University of Science and Technology, Hong Kong, PR China; 4 Laboratory for Genetics of Disease Susceptibility, Li Ka Shing Institute of Health Sciences, The Chinese University of Hong Kong, Hong Kong, PR China

## Abstract

**Background:**

The interactions of multiple single nucleotide polymorphisms (SNPs) are highly hypothesized to affect an individual's susceptibility to complex diseases. Although many works have been done to identify and quantify the importance of multi-SNP interactions, few of them could handle the genome wide data due to the combinatorial explosive search space and the difficulty to statistically evaluate the high-order interactions given limited samples.

**Results:**

Three comparative experiments are designed to evaluate the performance of MegaSNPHunter. The first experiment uses synthetic data generated on the basis of epistasis models. The second one uses a genome wide study on Parkinson disease (data acquired by using Illumina HumanHap300 SNP chips). The third one chooses the rheumatoid arthritis study from Wellcome Trust Case Control Consortium (WTCCC) using Affymetrix GeneChip 500K Mapping Array Set. MegaSNPHunter outperforms the best solution in this area and reports many potential interactions for the two real studies.

**Conclusion:**

The experimental results on both synthetic data and two real data sets demonstrate that our proposed approach outperforms the best solution that is currently available in handling large-scale SNP data both in terms of speed and in terms of detection of potential interactions that were not identified before. To our knowledge, MegaSNPHunter is the first approach that is capable of identifying the disease-associated SNP interactions from WTCCC studies and is promising for practical disease prognosis.

## Background

Single nucleotide polymorphisms (SNPs) are single nucleotide variations of DNA base pairs. Researchers often use SNPs as genetic markers in disease studies. It has been well established in the field that SNP profiles characterize a variety of diseases. By investigating SNP profiles associated with a disease trait, researchers would be able to reveal relevant genes. However, in many complex diseases, SNPs have shown little penetrance individually; on the other hand, their interactions are suspected to possess stronger associations with complex diseases. Some SNPs, which have no direct impact on health, may be linked to nearby genes which do have effects. Researchers hypothesize that many common diseases in humans are not caused by one genetic variation within a single gene, but are determined by complex interactions among multiple genes. Since the sheer volume of data generated by SNP studies is difficult to be manually analyzed, an efficient computational model is required to detect or indicate which pattern is most likely associated with the disease. Then, it will just be a matter of time before physicians can screen individuals for susceptibility to a disease by analyzing their DNA samples for specific SNP patterns, and further design some experiments to target the genes that implicate the disease.

Recently, many methods have been proposed to identify SNP interaction patterns associated with diseases. To name a few studies, BEAM [1] designed a Bayesian marker partition model and used MCMC sampling strategy to estimate the model parameters; MDR [2] applied an exhaustive search model to evaluate all possible multi-SNP interactions under some given thresholds; the penalized regression [3] used a variant of logistic regression model with quadratic penalization; CPM [4] used a combinatorial partitioning method for finding the interacted SNPs; RPM [5] extended CPM by using some heuristics to reduce the search space; Monte Carlo Logic Regression [6] combined the logic regression and MCMC in searching the SNP interactions; BGTA [7] proposed a screening algorithm to repeatedly evaluate a large number of randomly generated marker subsets. HapForest [8] used a forest-based approach to identifying haplotype-haplotype interactions. Although these methods perform well on small data sets, most of them (except BEAM) are unable to efficiently detect the multi-SNP interactions in genome wide association study.

BEAM has successfully demonstrated its capability of handling large data sets using synthetic data. When the authors applied BEAM to an AMD (aged-related macular degeneration) study [9], however, BEAM did not report any interactions. One possible reason is that the number of samples is not sufficient to detect the statistically significant interactions. Another possible reason is that BEAM treats local SNP interactions (haplotype effect) equally with global gene interactions during MCMC sampling, which could miss some critical haplotype effects in a genome wide association study because haplotype effects generally appear more frequently than global gene interactions.

Given a genome wide association study with thousands of SNPs and a limited number of samples, it is difficult to detect and evaluate the multi-SNP interactions in a traditional statistic manner. The feasible solution is to first find a small set of relatively more relevant SNPs and then evaluate the interactions within it. This procedure was applied in HapForest [8] to infer the haplotype-haplotype interaction.

However, the typical feature selection models, which use univariate ranking on feature importance and arbitrary threshold to select relevant features, cannot be applied because they will filter out those SNPs that have weak marginal effects, while their joint behavior may significantly contribute to disease traits. In this paper, we introduce an alternative learning approach (MegaSNPHunter) to hierarchically rank the multi-SNP interactions from local genomic regions to global genome. MegaSNPHunter takes case-control genotype data as input and produces a ranked list of multi-SNP interactions. In particular, the whole genome is first partitioned into multiple short subgenomes and each subgenome covers the genomic area of possible haplotype effects in practical. For each subgenome, MegaSNPHunter builds a boosting tree classifier based on multi-SNP interactions and measures the importance of SNPs one the basis of their contributions in the classifier. The method keeps relatively more important SNPs from all subgenomes and let them compete with each other in the same way at the next level. The competition terminates when the number of selected SNPs is less than the size of a subgenome. At the last step, MegaSNPHunter extracts and reports the valuable multi-SNP interactions.

## Results

The performance of MegaSNPHunter is evaluated through comparative studies with existing work. The goal of MegaSNPHunter is to discover the multi-SNP interactions from genome wide studies. Among many recently proposed methods, BEAM is the best one which could handle the large scale data set and finish in a reasonable time. Therefore, we mainly compare our method with BEAM in this paper using synthetic data generated on the basis of epistasis models and the data sets from two real studies on complex diseases. In the experiments on two real studies, one uses a genome wide study on Parkinson disease (data acquired by using Illumina HumanHap300 SNP chips [10]). The other experiment chooses the rheumatoid arthritis study [11] from Wellcome Trust Case Control Consortium (WTCCC) using Affymetrix GeneChip 500K Mapping Array Set. In our experiments, a SNP marker can take one of the following four states: 0 (missing), 1 (coding for the homozygous reference), 2 (heterozygous), and 3 (homozygous variant). The class label is either 0 (control) or 1 (case).

### Experiment on Simulation study

**Figure 1:**
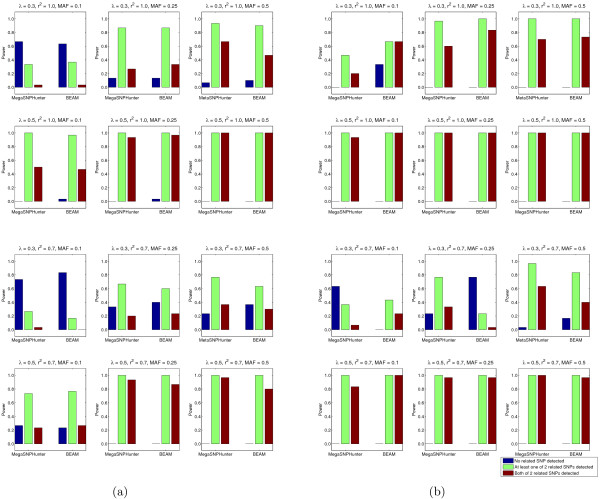
**Comparison between MegaSNPHunter and BEAM on synthetic data**. Comparison between MegaSNPHunter and BEAM on synthetic data. For each setting, the power is calculated as the proportion of 30 data sets. Each data set contains 2000 samples (1000 cases and 1000 controls) and 1000 SNPs. *λ *controls the marginal effect. MAF is the minor allele frequency. LD between each unobserved disease locus and the associated marker is measured by *r*^2^. (a): The performance comparison on additive model. (b):The performance comparison on multiplicative model.

Simulation studies are developed to validate the performance of our approach in correctly determining the associated SNPs defined by an epistatic model. To make the fair comparison, we use the simulation program provided in BEAM package and follow the same procedure in [1] to generate the data based on two epistatic models (additive effect and multiplicative effect). For each model, we choose 12 settings (readers may refer [1] for details) and for each setting, we generate 30 data sets, and each data set includes 1000 SNPs and contains 2000 samples (1000 cases and 1000 controls). The performances of both MegaSNPHunter and BEAM are illustrated in Figure 1. In most settings, MegaSNPHunter performs the same or slightly better than BEAM.

Ideally, the results on the genome wide simulation would be more convincing but such a simulation is computationally expensive. In general, the goal of simulation study is to provide the evidence for validity of our approach. In practice, the real data is very complex and the SNP interactions in the real data may not match any epistatic model. Therefore, our approach does not assume any epistatic model. We believe the most effective criterion for judging the epistatic interaction is that the joint effect is much more significant than the marginal effects of individual SNPs. The next two experiments would show the effectiveness of our approach on the real data.

### Experiment on Parkinson study

Parkinson disease is a chronic neurodegenerative disease with a cumulative prevalence of greater than 0.1 percent. The primary symptoms of Parkinson's disease include tremors, rigidity, slow movement, poor balance, and difficulty walking. In this experiment, we choose the study in [10] which provides around 396,000 genotypes in 541 samples. Both BEAM and MegaSNPHunter are tested on this data set. BEAM could not identify any interaction while our MegaSNPHunter selected 7 significant SNP interactions.

MegaSNPHunter is first run on each chromosome with 10 fold cross validation. Cross validation is a model evaluation method that estimates how well the model built from some training data is going to perform on unseen data. The 10 fold cross validation is conducted every time when the boosting tree classifier is built in the whole hierarchical procedure. In our test, the samples are randomly sampled into 10 subsets and each validation uses 9 subsets to train the model and the left one to test the performance. The output from every validation is a classifier and a list of ranked SNPs.

After 10 validations are finished, a post process is invoked to isolate those SNPs whose genotype association *χ*^2 ^*P *values reach a critical value (default is 0.05), and those SNPs whose interaction's genotype association *χ*^2 ^*P *values are above a critical value (default is 0.0025). The top ranked SNPs among the selected 302 SNPs are reported in Table 1 with genotype association *χ*^2 ^*P *values. The selected interactions with genotype association *χ*^2^*P *values are reported in Table 2. To handle the multiple test issue, we conduct an extra permutation-based test (chromosome level) on both single SNP and SNP interactions to correct P values.

**Table 1:** Identified SNPs for Parkinson study.

SNP reference	Chromosome	Genotype association *χ*^2 ^*P *value	Permutation test *P *value
rs6826751	4	7.647 * 10^-7^	2.0 * 10^-4^

rs4888984	16	1.351 * 10^-5^	6.0 * 10^-4^

rs2986574	1	1.402 * 10^-5^	6.0 * 10^-4^

rs1480597	10	1.862 * 10^-5^	0.0016

rs13032261	2	2.233 * 10^-5^	0.0012

rs546171	9	3.104 * 10^-5^	2.0 * 10^-4^

rs7554157	1	3.428 * 10^-5^	0.0010

rs999473	10	3.82 * 10^-5^	0.0022

rs7924316	11	3.883 * 10^-5^	6.0 * 10^-4^

rs2235617	20	4.656 * 10^-5^	8.0 * 10^-4^

rs13135430	4	5.805 * 10^-5^	0.0060

rs243023	2	6.90 * 10^-5^	0.0012

rs11691934	2	8.246 * 10^-5^	0.0022

This table reports the top ranked SNPs and their genotype association *χ*^2 ^*P *values.

**Table 2:** Selected interactions for Parkinson study.

Interacted SNPs	Genotype association *χ*^2 ^*P *value	Permutation test *P *value
rs2235617 ⇔ rs2470378	2.318 * 10^-7^	3.0 * 10^-6^

rs7172832 ⇔ rs906428	4.219 * 10^-7^	2.89 * 10^-4^

rs1505376 ⇔ rs3861561	4.998 * 10^-7^	1.62 * 10^-4^

rs13032261 ⇔ rs7924316	2.824 * 10^-6^	2.72 * 10^-4^

rs13032261 ⇔ rs2284967	6.325 * 10^-6^	3.39 * 10^-4^

rs13032261 ⇔ rs906428	6.402 * 10^-6^	3.44 * 10^-4^

rs842796 ⇔ rs800897	6.596 * 10^-6^	3.36 * 10^-4^

This table reports the selected interactions and their genotype association *χ*^2 ^*P *values.

**Figure 2:**
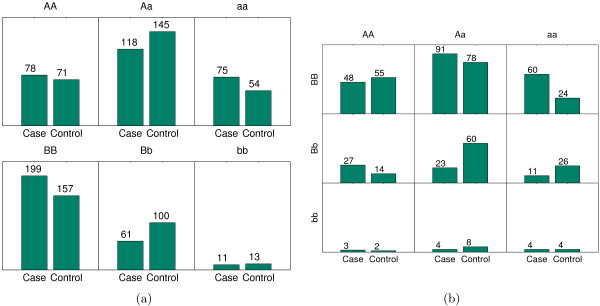
**The joint effect of rs7172832 and rs906428, and their marginal effects**. The joint effect of rs7172832 and rs906428, and their marginal effects. (a): The distribution of cases and controls of rs7172832 (*P *value 0.03) and rs906428 (*P *value 0.001); (b): The distribution of cases and controls under the interaction of rs7172832 and rs906428 (*P *value 4.219 * 10^-7^).

**Figure 3:**
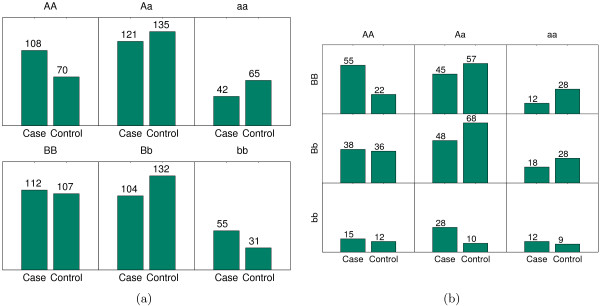
**The joint effect of rs1505376 and rs3861561, and their marginal effects**. The joint effect of rs1505376 and rs3861561, and their marginal effects. (a): The distribution of cases and controls for rs1505376 (*P *value 0.001) and rs3861561 (*P *value 0.012). (b): The distribution of cases and controls under the interaction of rs1505376 and rs3861561 (*P *value 4.998 * 10^-7^).

We observe that among 12 SNPs involved in the selected interactions in Table 2, only three of them (*rs*13032261, *rs*7924316 and *rs*2235616) have noticeable marginal effects in Table 1. For the other 9 SNPs, their joint effects are much more significant than the corresponding individual SNP effects. Figure 2 shows the genotype distribution of two SNPs (*rs*7172832 and *rs*906428) and the genotype distribution under the interaction. Figure 3 displays the same information for the interaction between *rs*1505376 and *rs*3861561. These figures clearly illustrate how the two weak SNPs significantly affect disease traits (the first interaction is not in this case because the marginal effect of *rs*2235617 is already significant).

### Experiment on rheumatoid arthritis study

**Figure 4:**
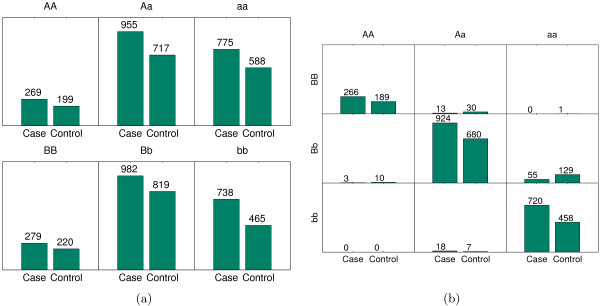
**The joint effect of rs4523817 and rs4418931, and their marginal effects**. The joint effect of rs4523817 and rs4418931, and their marginal effects. (a): The distribution of cases and controls for rs4523817 (*P *value 0.866) and rs4418931 (*P *value 0.001). (b): The distribution of cases and controls under the interaction of rs4523817 and rs4418931 (*P *value 6.83 * 10^-15^).

The Wellcome Trust Case Control Consortium (WTCCC) is a collaboration of many British research groups. To date, the WTCCC has examined the genetic signals of seven common human diseases: rheumatoid arthritis, hypertension, Crohn's disease, coronary artery disease, bipolar disorder, and type 1 and type 2 diabetes. The rheumatoid arthritis study [11] contains around 500 *K *genotypes in 3503 samples (1999 cases and 1504 controls). We use the same procedure mentioned above to conduct the experiment. The top ranked SNPs among the selected 213 SNPs are reported in Table 3 with genotype association *χ*^2 ^*P *values. The selected interactions with genotype association *χ*^2 ^*P *values are reported in Table 4. The top interaction identified in MegaSNPHunter is between *rs*4418931 and *rs*4523817. Its genotype association *χ*^2 ^*P *value is 6.83 * 10^-15^. The genotype distribution of cases and controls for these two SNPs and the distribution under their interaction are plotted in Figure 4.

**Table 3:** Identified SNPs for WTCCC study.

SNP reference	Chromosome	Genotype association *χ*^2 ^*P *value	Permutation test *P *value
rs17163819	2	2.587 * 10^-150^	0.0042

rs10894818	12	1.751 * 10^-120^	0.0046

rs582397	3	1.089 * 10^-82^	0.0022

rs7596121	3	5.212 * 10^-60^	0.0022

rs16898558	6	1.718 * 10^-52^	0.0046

rs996877	13	1.566 * 10^-44^	0.0036

rs9387380	7	2.315 * 10^-34^	0.011

rs940153	9	1.032 * 10^-33^	0.0040

rs1456222	4	1.544 * 10^-33^	0.0048

rs1572075	5	1.474 * 10^-23^	0.0040

rs7192563	17	2.862 * 10^-18^	0.0030

rs17765376	15	3.277 * 10^-18^	0.0058

rs9532645	14	1.26 * 10^-16^	0.0028

rs10751815	11	1.036 * 10^-15^	0.0014

rs6975106	8	3.207 * 10^-13^	0.0028

This table reports the top ranked SNPs and their genotype association *χ*^2 ^*P *values.

**Table 4:** Selected interactions for WTCCC study.

Interacted SNPs	Genotype association *χ*^2 ^*P *value	Permutation test *P *value
rs4418931 ⇔ rs4523817	6.83 * 10^-15^	0.001382

rs6696928 ⇔ rs10493711	2.075 * 10^-12^	0.00216

rs262714 ⇔ rs407818	6.532 * 10^-8^	0.00240

rs2041377 ⇔ rs11113207	6.95 * 10^-8^	0.00236

rs7459039 ⇔ rs10271302	1.073 * 10^-8^	0.003224

rs17565060 ⇔ rs7220740	3.406 * 10^-7^	0.00345

rs9268230 ⇔ rs7751204	6.90 * 10^-7^	0.0112

rs17507967 ⇔ rs12126069	8.622 * 10^-7^	0.00384

rs3738369 ⇔ rs11206109	1.53 * 10^-6^	0.00389

This table reports the selected interactions and their genotype association *χ*^2 ^*P *values.

Both *rs*4418931 and *rs*4523817 are located on the gene *GPC*6, which is a member of the glypican gene family and encodes a product structurally related to *GPC*4 [12]. In a latest study of rheumatoid arthritis [13], *GPC*4 displays strong expression. The connection between our finding and previous work may imply a complex rheumatoid arthritis associated pattern. More evidences from biological aspect are under investigation. Again, BEAM could not report any significant interaction. The reason that BEAM could not report any interaction is partly because the data from the real studies are too complex to be formulated by one Bayesian marker partition model and the distribution assumptions in BEAM may not be true for the real data. The results from both experiments on real data sets empirically justify that our method performs better than BEAM with respect to finding SNP interactions in genome wide association studies.

### Running time comparison

Another attracting point of our MegaSNPHunter is that it runs faster than BEAM. Suppose the number of SNPs in each subgenome is *W*, the number of SNPs is *M*, and the number of samples is *N*. Then the number of subgenomes is $\frac{M}{W}$ + 1. The time for training one boosting tree classifier using one subgenome is *O*(*W *· *N *· log(*N*)). Then the time for learning at the first level is *O*(*M *· *N *· log(*N*)). The expected number of SNPs at the second level is $\frac{M}{2}$, and $\frac{M}{2^{d-1}}$ at the *d*_*th *_level. Then the time for the learning at the *d*_*th *_level is *O*($\frac{M}{2^{d-1}}$ · *N *· log(*N*)). The total running time is *O*(*M *· (1 + $\frac{1}{2}$ + ⋯ + $\frac{1}{2^{2d-1}}$) · *N *· log(*N*)) that is equivalent to *O*(*M *· *N *· log(*N*)). It approximates to 6.20 * 10^9 ^for the rheumatoid arthritis study, which is much less than the complexity *O*(I * *N*) (around 3.5 * 10^11^) of BEAM, where *I *is the number of iterations in MCMC sampling and is set to 10^8^as default value for a data set with medium size (i.e. around 400, 000 SNPs). Theoretically, *I *is determined by *O*(M * *N*^*d*^) with *d *denoting the number of interacting SNPs (i.e. interaction depth).

### Discrimination ability on real data sets

As for the discrimination power of MegaSNPHunter, Table 5 and Table 6 report the prediction accuracies for both experiments on real data sets. They also report the prediction accuracies for each chromosome based on selected SNPs and the prediction accuracies from randomized tests for comparison. The randomized tests randomly select the same number of SNPs as our method has selected for each chromosome and the whole genome, and collect the prediction accuracies using 10-fold CV. The reported accuracies for randomized tests are the averages of 50 runs. In both tables, we observe that the randomly selected SNPs from both real data sets can only achieve around 50% prediction accuracy on average. We realize that there are many false positives in selected SNPs because MegaSNPHunter can achieve good performance on every chromosome. How to reduce the false positive error is a challenging problem in genome wide association studies. Although our method does not directly address this issue, nevertheless our method is able to reduce the number of possibly disease-associated SNPs and rank those SNPs based on their relevances to the disease trait. Extra filters can be applied to remove false positives.

**Table 5:** Classification for Parkinson study.

Chromosome	Picked SNPs	Total SNPs	Prediction Accuracy	Randomized test accuracy
1	242	31,532	0.852	0.505

2	247	32,706	0.874	0.516

3	218	27,691	0.874	0.517

4	174	24,193	0.835	0.511

5	188	24,570	0.878	0.507

6	204	26,372	0.857	0.501

7	278	21,382	0.821	0.498

8	254	22,434	0.845	0.508

9	243	19,542	0.841	0.505

10	227	20,007	0.841	0.507

11	247	19,539	0.854	0.513

12	230	19,572	0.806	0.506

13	156	14,123	0.784	0.502

14	224	12,645	0.824	0.509

15	212	11,618	0.786	0.518

16	225	11,767	0.793	0.496

17	202	11,619	0.778	0.507

18	252	12,613	0.793	0.507

19	165	8,608	0.802	0.5

20	186	10,375	0.806	0.512

21	130	6,612	0.758	0.497

22	126	7,071	0.782	0.506

OVERALL	339	396,588	0.913	0.503

The classification performance of MegaSNPHunter on Parkinson study.

**Table 6:** Classification for WTCCC study.

Chromosome	Picked SNPs	Total SNPs	Prediction Accuracy	Randomized test accuracy
1	154	39,428	0.947	0.512

2	109	40,641	0.968	0.565

3	153	33,121	0.932	0.523

4	127	31,343	0.926	0.486

5	151	31,601	0.905	0.498

6	130	31,133	0.915	0.546

7	126	25,412	0.938	0.553

8	109	26,954	0.927	0.523

9	143	23,246	0.905	0.552

10	125	28,222	0.881	0.482

11	132	26,005	0.905	0.516

12	113	24,721	0.887	0.492

13	86	18,913	0.896	0.504

14	94	15,436	0.865	0.511

15	112	14,192	0.911	0.504

16	115	15,070	0.903	0.532

17	101	11,128	0.887	0.513

18	135	14,633	0.893	0.522

19	85	6,286	0.885	0.540

20	106	12,266	0.874	0.503

21	80	7,014	0.892	0.496

22	76	6,124	0.924	0.533

OVERALL	223	451,288	0.926	0.513

The classification performance of MegaSNPHunter on WTCCC study.

### The parameter setting of MegaSNPHunter

There are four main parameters in the models, including the depth of trees, the threshold for selecting SNPs from trees, the subgenome size and the overlap between subgenome.

1. The depth of trees indicates the depth of SNP interaction. Since most significant interactions are depth 2, so as long as the depth of trees is above 2, the results would not be changed. MegaSNPHunter uses 5 as default setting.

2. The size of subgenome depends on the density of SNP data. Each subgenome should cover the genomic area of possible haplotype effects in practical. Before we start the experiment, we collect some statistics on how many SNPs are genotyped for one gene. This number will be used as the size of subgenome.

3. The overlap between subgenomes is used to solve the boundary problem between genes. Half of the size of subgenome is the best choice. Both the size of subgenome and the overlap between subgenomes depend on the priori knowledge on epistatic interactions.

4. The threshold for selecting SNPs from trees is a very critical parameter to the method. Our goal is to find interactions among SNPs with weak marginal effects. If the threshold is too stringent, then too many SNPs will be filtered out, while the loose threshold will allow too many SNPs to be selected. In our method, two strategies are applied to deal with this issue.

• The first strategy is to select all SNPs involved in the classifier. This is usually used in the situation where most SNPs are clearly irrelevant with diseases. However, in the worst case, the classifier may use all SNPs in training. If too many SNPs are selected in the classifier, the second strategy will be applied.

• The second strategy uses a threshold to select relevant SNPs. This threshold is the critical value of *χ*^2 ^statistic. The default setting for single SNP is 0.05, 0.05*0.05 for a pair of interacted SNPs, and so on so forth.

## Discussion

### The advantages of MegaSNPHunter

The development of MegaSNPHunter was triggered by the limitations of existing works on finding high order SNP interactions from genome wide studies. Given a genome wide study containing thousands of markers, most existing methods either fail to report the statistically significant interactions due to the limited samples, or can not terminate in a reasonable time due to the explosive search space.

MegaSNPHunter addresses these issues by hierarchically reducing the number of relevant SNPs and then extracting the interactions. MegaSNPHunter displays many advantages over the existing methods:

• the hierarchical learning strategy can extract both local SNP interactions and global gene interactions in an efficient manner without exhaustive enumeration;

• MegaSNPHunter uses a classifier built on SNP interactions to rank the relevances of SNPs, which is superior to the univariate feature selection techniques on finding the SNPs with weak marginal effects but significant joint effects;

• MegaSNPHunter is a non parametric method. It does not assume any prior distributions as required by many parametric-statistical methods;

• MegaSNPHunter does not assume any particular epistasis models, which is very important for real studies because the models of SNP interactions are unknown and likely to be very complex. Our method only assumes that the further the distance between two SNPs, the less possibility they interact with each other.

• MegaSNPHunter could be applied for discrimination, where we can use the selected SNPs to build a classifier for discriminating two or more classes of samples.

### The limitations of MegaSNPHunter

The big advantage of MegaSNPHunter is to find the interactions between SNPs with weak marginal effects. To handle the high dimension of genome wide data, MegaSNPHunter partitions the whole genome into multiple short subgenomes and select the relative more important SNPs from each subgenome. If the interacted SNPs are not located in the same subgenome, MegaSNPHunter requires that their marginal effects must be above the medium of marginal effects of their resided subgenomes. We think this is a soft constraint because in reality, most SNPs in the genome do not contribute to any trait variation. If either of interacted SNPs only has trivial marginal effect, it would have little chance to survive and meet its counterpart in the next level.

In the real application, MegaSNPHunter could incorporate some search strategies proposed in [14] as a preprocess to reduce the search space. These search strategies first find disease-associated SNPs with noticeable marginal evidence. Then an exhaustive search procedure can be applied to find interactions among them. These strategies complements our method. We could start from using them to find interactions between SNPs with strong marginal effects and next run MegaSNPHunter to find interactions between SNPs with weak marginal effects.

### Future Studies

There are several issues we need to address in the future work. Since our method assumes that the strength of interaction is inversely proportional to the distance of SNPs, most findings in our results are local effects. The interactions between SNPs far in distance have already drawn many researchers' attention. We plan to develop new methods to find the global SNP interactions. An efficient sampling strategy is one possible solution. Another critical issue is how to reduce false positives. We plan to incorporate the haplotype information and pathway information to help reduce the false positive error in future study.

## Conclusion

In this paper, we propose a novel hierarchical learning algorithm (MegaSNPHunter) to find high order SNP interactions in genome wide association studies. We evaluate MegaSNPHunter through comparative studies on simulated data and the data sets from two real studies including a genome wide study on Parkinson disease [10] and the rheumatoid arthritis study from WTCCC [11]. In the simulation experiment, MegaSNPHunter displays the comparable performance while in the experiments on two real studies, BEAM could not report any interaction patterns but our MegaSNPHunter identifies many interactions among SNPs whose joint effects are more significant than the individual SNP effects. In summary, the hierarchical nature of our non-parametric learning scheme enables our new method to search for interaction patterns more efficiently than existing methods. In this sense, our method is a powerful tool for whole genome data analysis.

## Methods

**Figure 5:**
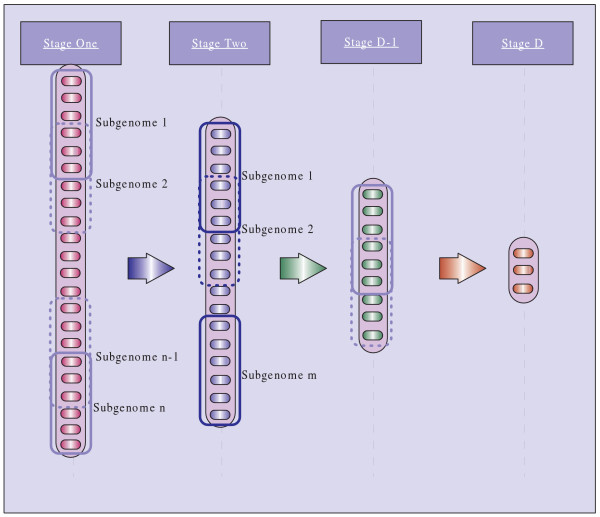
**The flowchart of MegaSNPHunter**.

The goal of MegaSNPHunter is to find the remarkable multi-SNP interactions from large genome data to explain the observed trait variation. To handle the high dimension of genome wide data, MegaSNPHunter adopts a hierarchial learning approach that first reduces the number of relevant SNPs into a small set and then extract the multi-SNP interactions. In the process of finding relevant SNPs, the whole genome is first divided into multiple short subgenomes, and the next step is to rank the importance of SNPs by building a classifier with multi-SNP interactions for each subgenome. The importance of SNPs in each classifier is measured by their contributions to the classification power. The flowchart of MegaSNPHunter is illustrated in Figure 5. In the following sections, the base learner for each subgenome is introduced first. Next, the hierarchical learning algorithm is described in details. At last, a new procedure different from brute-force search is presented to extract the multi-SNP interactions from tree classifiers.

### Tree Boosting Classifier

There are many popular classification models in machine learning, which could be chosen as our base learner. Among them, classification and regression tree (CART) [15] is one of the best choices because the tree based learning model has a good interpretability of feature interaction. CART recursively generates a tree model by splitting the data using selected features. It uses the GINI index to determine how well the splitting rule separates samples contained in the parent node. Once the best split is found, CART repeats the splitting process for another child node, and continues recursively until further splitting is impossible. The interaction of features is represented as a path from the root node to the leaf node in the tree. However, the tree-based model is usually not stable and often sensitive to the data distribution. To increase its discrimination power, one popular solution is to use boosting [16]. Boosting is considered as one of the most powerful learning procedure that theoretically could be used to boost any weak learner (even only slightly better than a random guess), and combine a set of weak learners into a strong learner. Among all boosting models, gradient boosting of regression tree [17] is considered as a highly robust and competitive method for feature selection. It shows excellent performance even when the number of features is large and the relationship between features and class is complex. The general gradient boosting procedure [17] is listed in Algorithm 1 (shown at the end of the paper). The basic idea is to compute a sequence of regression trees, where each successive tree is built for the prediction residuals of the preceding tree. To avoid the overfitting, the size of the trees is usually fixed to some pre-given threshold. *L*(*Y*, *f*(*X*)) in Algorithm 1 is the loss function to minimize. For a two-class classification in boosting, the loss function is the negative binomial log-likelihood defined in [17] as

(1) *L*(*y*, *f*) = -∑(*y*_*i*_*f*(*x*_*i*_) - log(1 + exp(*f*(*x*_*i*_)))), *y *∈ {0,1},

where *f*(*x*) is defined as

(2) $$f(x)=\log[\frac{P(y=0|x)}{P(y=1|x)}].$$

The gradient of loss function *L*(*Y*, *f*(*X*)) is derived as

(3) $$z_{i}=y_{i}-\frac{1}{1+exp(-f(x_{i}))}.$$

The output *F *of this procedure is a set of regression trees that are added together to perform the classification task.

**Algorithm 1 **General Gradient Boosting Procedure

   Initialized F to be a constant.

   **for ***t *= 0 to *T ***do**

      Compute the negative gradient *z*_*i *_= -$\frac{\partial }{\partial f(x_{i})}$*L*(*y*_*i*_, *f*(*x*_*i*_))

      Fit a regression tree T(x), predicting *z*_*i*_

      Update F as *F *← *F *+ *ηT*(*x*)

   **end for**

### MegaSNPHunter

MegaSNPHunter takes case-control genotype SNP data as input and produces a ranked list of multi-SNP interactions. To find non-trivial multi-SNPs interactions in the high dimension of genome wide data, a general approach would first evaluate each SNP individually and select some top ranked ones, and then extract the multi-SNP interactions in the selected SNPs. This approach falls short at finding those significant interactions among SNPs with weak marginal effects because those SNPs have high probabilities to be filtered out in the first step. Taking multi-SNP interactions into account in the selection stage provides a good solution to this issue. MegaSNPHunter employs a hierarchial learning strategy. In particular, the whole genome is first divided into multiple short subgenomes and a tree boosting classifier is built on each subgenome. The built classifier consists of a collection of regression trees, where each node represents one SNP and each path in the trees indicates a possible interaction of those SNPs on the path. Given a tree boosting classifier {*T*_*j*_}, the importance of each SNP is measured by its classification contribution to the classification power, which is defined as

(4) $$I(S_{i})=\frac{1}{J}\sum_{j=1}^{J}\sum_{v\in T_{j}}e_{v}1(v=S_{i}),$$

where *e*_*v *_is the empirical error reduction by splitting on *x*_*i *_using SNP *S*_*i *_in tree *T*_*j *_[18]. The average of the relative influence of SNP *S*_*i *_across all the trees is used to measure its importance.

Using Equation 4, MegaSNPHunter could rank the importance of SNPs in each subgenome. A cut-off threshold can be used to choose the top ones. The selected SNPs from all subgenomes will first merge together and then compete with each other in the same way at the next level. By having all SNPs compete with each other in training classifiers, MegaSNPHunter reduces the large number of relevant SNPs into a very small set. For this small set of SNPs, the multi-SNP interactions could be extracted and ranked even using the brute-force search method like MDR. Nevertheless, one critical drawback of MDR lies in the places that the search depth, which is equivalent to the order of SNP interaction, has to be limited to some certain level in order to complete the search in a reasonable time. In MegaSNPHunter, we design a new procedure to extract the high orders of multi-SNP interactions without exhaustive enumeration.

### Interaction Extraction

Given a small set of SNPs, it is feasible to test all possible interactions using exhaustive search. However, the number of selected SNPs from a genome wide study may still make exhaustive search of high order interactions very time consuming. Concretely, the number of possible interaction for *n *SNPs with maximal depth *d *is $C_{n}^{2}+C_{n}^{3}+\cdots +C_{n}^{d}$. For example, 50 SNPs with maximal depth 5 would give rise to 2,369,885 possible SNP interactions, which would go much higher even with a small increase on the number of SNPs or the maximal depth of SNP interactions. Apparently, the brute-force search method for extracting high orders of SNP interactions is not a good choice in MegaSNPHunter. In MegaSNPHunter, the built classifier is a collection of trees in which each path represents a possible interaction among SNPs on the path. For those SNP interactions making non-trivial contribution to the traits (case or control) of samples, it is very likely that they will be included in the boosting classifier. Therefore, we could first extract all possible paths from trees and then evaluate the interactions of SNPs on each path. Given *K *binary trees with maximal depth *d*, the number of paths from root nodes to leaf nodes is K * 2^*d*-1^. For each length *d *path from the root node to the leaf node, the number of possible sub-paths with length at least 2 is $\frac{\left(d-1)(d-2\right)}{2}$. Then the total number of possible interactions in our procedure is K * 2^*d*-2 ^* (*d *- 1) (*d *- 2) which is far less than $C_{n}^{2}+C_{n}^{3}+\cdots +C_{n}^{d}$ (*n *is the number of SNPs) for a brute-force search.

After extracting all possible SNP interactions from the classifier, we rank them using the H-statistics proposed in [18]. For two given variables (*x*_*j*_, *x*_*k*_), the H statistic is defined as

(5) $$H(x_{j},x_{k})=\frac{\sum_{i=1}^{N}{\left[{\hat{F}}_{jk}(x_{ij},x_{ik})-{\hat{F}}_{j}(x_{ij})-{\hat{F}}_{k}(x_{ik})\right]}^{2}}{\sum_{i=1}^{N}{\hat{F}}_{jk}^{2}(x_{ij},x_{ik})},$$

where ${\hat{F}}_{s}$({*x*_*j*_}_*j*∈*s*_) estimates the partial dependence of the classifier *F *on {*x*_*j*_}_*j*∈*s*_, which is defined as

(6) $${\hat{F}}_{s}({\left\{x_{j}\right\}}_{j\in s})=\frac{1}{N}\sum_{i=1}^{N}F({\left\{x_{j}\right\}}_{j\in s},{\left\{x_{ik}\right\}}_{k\notin s}).$$

The partial dependence ${\hat{F}}_{s}$({*x*_*j*_}_*j*∈*s*_) is equivalent to the marginal effect of {*x*_*k*_}_*k *∉ *s *_in classifier *F*. Therefore, *H*(*x*_*j*_, *x*_*k*_) measures the fraction of partial dependence ${\hat{F}}_{jk}$(*x*_*j*_, *x*_*k*_) not captured by ${\hat{F}}_{j}(x_{j})+{\hat{F}}_{k}(x_{k})$. The H-statistics of high order interactions are defined in the same way as in [18].

### Algorithm

To summarize, we propose the hierarchical learning algorithm 2.

**Algorithm 2 **MegaSNPHunter Algorithm

   **Given:**

   D:the depth of interactions

   W:the subgenome size

   L:The overlap size

   S:SNP Data [*X*, *Y*] with class label.

   **while ***numberOfSNPs*(*S*) > *W ***do**

      *SelectedSNPs *← ∅

      Separate *S *into *S*_0_, *S*_1_,...,*S*_*m *_where *sizeof*(*S*_*i*_) = *W *(*i *<*m*), *sizeof*(*S*_*i *_∩ *S*_*j*_) = *O *and *sizeof*(*S*_*m*_) <*W*

      **for ***i *= 0 to *m ***do**

         *F*_*i *_← *TreeBoostingClassfier*(*S*_*i*_, *T*, *D*)

         **for ***SNP*_*j *_∈ *F*_*i *_**do**

            *SelectedSNPs *← *SelectedSNPs *+ {*SNP*_*j*_}

         **end for**

      **end for**

      *S *← *S*(*SelectedSNPs*)

   **end while**

   *F *← *TreeBoostingClassfier*(*S*, *T*, *D*).

   Extract all path *P*_*i *_from *F*.

   Compute H-statistic *H*(*P*_*i*_) and Rank *P*_*i*_.

   **Function ***T reeBoostingClassfier*([*X*, *Y*], *T*, *D*)

   *F *← 0

   **for ***t *= 0 to *T ***do**

      *e*_*i *_= *y*_*i *_-$\frac{1}{1+exp(-F(x_{i}))}$, *i *∈ (1, *n*)

      Fit a *D *depth regression tree ${\left\{R_{l}\right\}}_{L}^{1}=E({\left\{R_{l}\right\}}_{L}^{1}|e_{i})$

      $\lambda_{l}=\frac{\sum \left(y_{i}-{\tilde{y}}_{i}\right)}{\sum {\tilde{y}}_{i}(1-{\tilde{y}}_{i})}$ where ${\tilde{y}}_{i}=\frac{1}{1+exp(-F(x_{i}))}$

      *F *← *F *+ *η*∑*λ*_*l*_1(*x *∈ *R*_*l*_)

   **end for**

   **return ***F*

## Authors' contributions

X.W. and C.Y. designed the models and simulation studies together. N.T. and W.Y. initialized the study and proposed the framework of multi-level learning. Q.Y. and H.X. direct the evaluation of methodologies. All authors contributed to the writing of the manuscript.

## References

[B1] Zhang Y, Liu JS (2007). Bayesian inference of epistatic interactions in case-control studies. Nature Genetics.

[B2] Ritchie MD, Hahn LW, Roodi N, Bailey LR, Dupont WD, Parl FF, Moore JH (2001). Multifactor-dimensionality reduction reveals high-order interactions among estrogen-metabolism genes in sporadic breast cancer. Am J Hum Genet.

[B3] Park MY, Hastie T (2008). Penalized logistic regression for detecting gene interactions. Biostatistics.

[B4] Nelson MR, Kardia SL, Ferrell RE, Sing CF (2001). A combinatorial partitioning method to identify multilocus genotypic partitions that predict quantitative trait variation. Genome Research.

[B5] Culverhouse R, Klein T, Shannon W (2004). Detecting epistatic interactions contributing to quantitative traits. Genetic Epidemiology.

[B6] Kooperberg C, Ruczinski I (2005). Identifying interaction SNPs using Monte Carlo logic regression. Genetic Epidemiology.

[B7] Zheng T, Wang H, Lo SH (2006). Backward genotype-trait association (BGTA) – based dissection of complex traits in case-control design. Hum Hered.

[B8] Chen X, Liu CT, Zhang M, Zhang H (2007). A forest-based approach to identifying gene and gene-gene interactions. PNAS.

[B9] Robert JK, Caroline Z, Emily YC, Jen-Yue T, Richard SS, Chad H, Alice KH, John PS, Shrikant MM, Susan TM, Michael BB, Frederick LF, Jurg O, Colin B, Josephine H (2005). Complement factor H polymorphism in age-related macular degeneration. Science.

[B10] Fung H, Scholz S, Matarin S, Simn-Snchez S, Hernandez D, Britton A, Gibbs J, Langefeld C, Stiegert M, Schymick J (2006). Genome-wide genotyping in Parkinson's disease and neurologically normal controls: first stage analysis and public release of data. Lancet Neurol.

[B11] The Wellcome Trust Case Control Consortium (2007). Genome-wide association study of 14,000 cases of seven common diseases and 3,000 shared controls. Nature.

[B12] Stephenie PS, Beth LV, Scott S (1999). GPC6, a Novel Member of the Glypican Gene Family, Encodes a Product Structurally Related to GPC4 and Is Colocalized withGPC5on Human Chromosome 13. Genomics.

[B13] Patterson AM, Cartwright A, David G, Fitzgerald O, Bresnihan B, Ashton BA, Middleton1 J (2008). Differential expression of syndecans and glypicans in chronically inflamed synovium. Ann Rheum Dis.

[B14] Marchini J, Donnelly P, Cardon LR (2005). Genome-wide strategies for detecting multiple loci that influence complex diseases. Nat Genet.

[B15] Breiman L, Friedman JH, Olshen RA, Stone CJ (1984). Classification and Regression Tree.

[B16] Schapire RE (1999). Theoretical views of boosting. Computational Learning Theory: Fourth European Conference, EuroCOLT.

[B17] Friedman JH, Hastie T, Tibshirani R (2000). Additive Logistic Regression: A statistical View of Boosting. Annals of Statistics.

[B18] Friedman JH, Popescu BE (2005). Predictive Learning via Rule Ensembles, Technical Report.

